# Prevalence and Molecular Characterization of *Cryptosporidium* spp., *Giardia duodenalis*, and *Enterocytozoon bieneusi* in Diarrheic and Non-Diarrheic Calves from Ningxia, Northwestern China

**DOI:** 10.3390/ani13121983

**Published:** 2023-06-14

**Authors:** Haihui Gao, Gaoxing Liang, Na Su, Qirui Li, Dong Wang, Jiandong Wang, Long Zhao, Xiaodong Kang, Kangkang Guo

**Affiliations:** 1 College of Veterinary Medicine, Northwest A&F University, Yangling 712100, China; gao6600022@163.com (H.G.); lgx20220425@163.com (G.L.); 18224792700@163.com (N.S.); lqr365698@126.com (Q.L.); xjau-wangdong@foxmail.com (D.W.); zhaolong4480@163.com (L.Z.); 2 Institute of Animal Science, Ningxia Academy of Agriculture and Forestry Sciences, Yinchuan 750002, China; jiandongwang668@126.com

**Keywords:** *Cryptosporidium* spp., *G. duodenalis*, *E. bieneusi*, prevalence, calf, diarrheic

## Abstract

**Simple Summary:**

This study first reported the different occurrences of *Cryptosporidium* spp., *G. duodenalis*, and *E. bieneusi* infection between diarrheic and non-diarrheic calves in Ningxia, China. The *Cryptosporidium* spp. infection rates were 54.0% and 38.3% in diarrheic and non-diarrheic calves, which was an extremely significant difference (*p <* 0.01). However, there was no statistical correlation between the prevalence of *G. duodenalis* infection as well as *E. bieneusi* infection and calf diarrhea (*p >* 0.05). Four *Cryptosporidium* species (*C. parvum*, *C. bovis*, *C. ryanae*, and *C. andersoni*), an assemblage E of *G. duodenalis*, and four *E. bieneusi* genotypes (BEB4, N, J, and NX1) were observed. The results showed that *Cryptosporidium* spp. is closely associated with calf diarrhea, and the identification of zoonotic species/assemblage/genotypes in calves suggests that these animals may play an important role in the transmission of zoonosis.

**Abstract:**

*Cryptosporidium* spp., *Giardia duodenalis*, and *Enterocytozoon bieneusi* are significant parasitic gastrointestinal pathogens with global distribution in humans and domestic animals, including calves. The main symptoms of calf infection are severe diarrhea, dehydration, growth retardation, and sometimes even death. To date, there has been limited information on the prevalence of *Cryptosporidium* spp., *G. duodenalis*, and *E. bieneusi* infections in calves in Ningxia, China, especially between diarrheic and non-diarrheic calves. A total of 438 fecal samples were collected from diarrheic (201) and non-diarrheic (237) calves in Ningxia. PCR and DNA sequencing were used to find the prevalence of *Cryptosporidium* spp. at 46.8% (205/438), *G. duodenalis* at 16.9% (74/438), and *E. bieneusi* at 10.0% (44/438). The prevalence of *Cryptosporidium* spp. infection in diarrheic and non-diarrheic calves was 54.0% (128/237) and 38.3% (77/201), respectively, and statistical analysis showed a positive correlation between the prevalence of *Cryptosporidium* spp. infection and calf diarrhea (*p* < 0.01). However, in this study, there was no statistical correlation between the prevalence of *G. duodenalis* infection as well as *E. bieneusi* infection and calf diarrhea (*p >* 0.05). Furthermore, four known *Cryptosporidium* species were successfully identified by comparing them with SSU rRNA gene sequences, including *C. parvum*, *C. bovis*, *C. ryanae,* and *C. andersoni*. In addition, all 74 *G. duodenalis*-positive samples were identified as assemblage E by comparative analysis of *bg* gene sequences. Among the 44 *E. bieneusi*-positive samples sequenced in the present study, 4 distinct *E. bieneusi* genotypes were successfully identified by comparative analysis of ITS sequences, including 3 known genotypes (J, BEB4, and N) and 1 novel genotype, the latter of which was identified and designated as NX1. These findings indicated that the high genetic diversity and complex population structures of *Cryptosporidium* spp., *G. duodenalis*, and *E. bieneusi* in Ningxia diarrhea calves and non-diarrhea calves, which provide new data for understanding the epidemiological status of *Cryptosporidium* spp., *G. duodenalis*, and *E. bieneusi* in Ningxia calves.

## 1. Introduction

Calf diarrhea is one of the most common and significant diseases in cattle, leading to serious economic losses to the development of the cattle industry due to therapeutic costs, labor, poor production performance, and deaths [1,2]. In the report of the 2007 National Animal Health Monitoring System (NAHMS) for U.S. dairy, 57% of weaned calves died from diarrhea [1]. The main symptoms of calf diarrhea are severe diarrhea, dehydration, vomiting, emaciation, growth retardation, and sometimes even death. Although there are scientific preventive measures for neonatal calf diarrhea, it still leads to high morbidity and mortality [3]. There are many reasons why diarrhea can develop in calves, including non-infectious factors such as genetics, age, herd and farm environment, nutrition, poor management, and other complications, as well as pathogenic factors such as bacteria, viruses, and parasites [4,5]. Usually, *Cryptosporidium* spp., *Giardia duodenalis*, and *Enterocytozoon bieneusi* are ubiquitous protozoan parasites that infect a broad range of vertebrate hosts including domestic and wild animals as well as humans, especially in children, neonatal animals, and immunocompromised individuals [5,6,7,8].

Molecular characteristics of the genotype and species of *Cryptosporidium* spp., *G. duodenalis*, and *E. bieneusi* are critical for the accurate identification and evaluation of zoonotic transmission [9]. To date, at least 44 valid species of *Cryptosporidium* spp. have been described, and at least 20 species and 5 genotypes have been detected in humans, indicating their zoonotic character [9,10,11]. The most common *Cryptosporidium* species in cattle are *C. ryanae*, *C. andersoni*, *C. parvum*, and *C. bovis* [10,11,12,13]. In addition, several other species have been described in a small number of cattle. *G. duodenalis* consists of eight distinct assemblages (A-H), and zoonotic assemblages A and B are infectious in both humans and animals [14]. The most common assemblages of *G. duodenalis* in cattle are E and A [15,16]. Over 600 *E. bieneusi* genotypes have been subdivided based on nucleotide divergence within the ITS rRNA gene fragment in animals and humans [17]. Phylogenetic analysis of these genotypes comprises 11 major groups [18]. Among them, group 1 is the largest category which comprises the majority of genotypes isolated from various hosts, including humans, and group 2 was previously thought to be a ruminant-specific genotype, but some of these genotypes also exist in humans [16,17,18], which were zoonotic. Groups 3–11 are mostly animals adapted [18]. Cattle are the potential hosts of *E. bieneusi* transmission, while their public health risks have always been neglected due to their scarce incidence rate in humans.

The fecal–oral route is the main transmission pathway of the three pathogens, and infection can also be spread by contaminated water, food, and fomites [19,20]. It is worth noting that an important source of contamination of water and food is cattle manure. Identification of *Cryptosporidium* spp., *G. duodenalis*, and *E. bieneusi* infection situation in cattle is essential to prevent and control the prevalence of these parasites. Previous studies to investigate *Cryptosporidium* spp., *G. duodenalis* and *E. bieneusi* in Ningxia used samples that were only from non-diarrheic adult cattle [21,22]. In this study, the prevalence and species of *Cryptosporidium* spp., *G. duodenalis,* and *E. bieneusi* among diarrheic and non-diarrheic calves in Ningxia, China were determined for the first time.

## 2. Materials and Methods

### 2.1. Sample Collection

**Figure 1:**
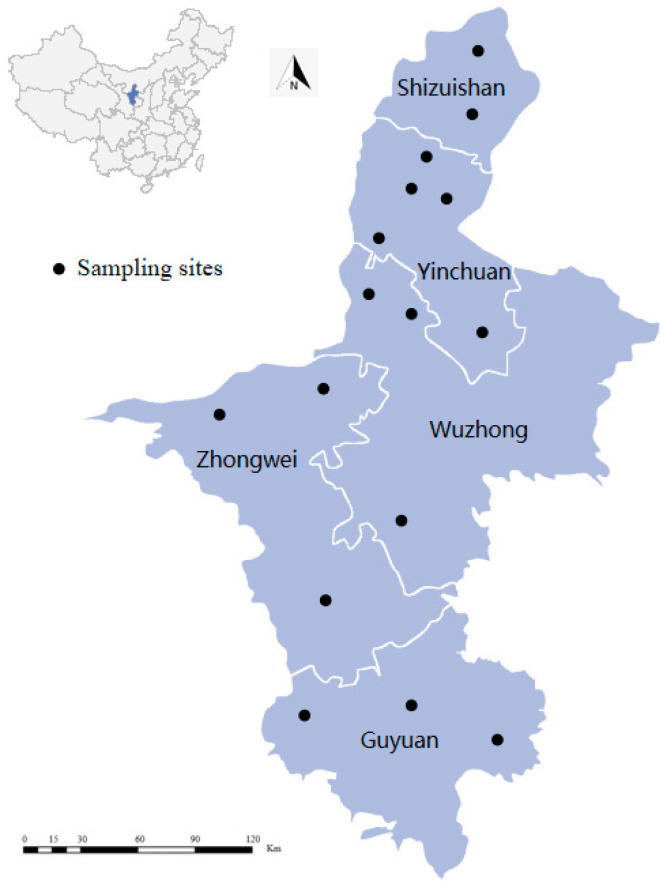
Sampling sites in Ningxia, China.

Ningxia is one of the important provinces in northern China to raise cattle. Because of environmental pollution and disease epidemics in traditional calf farming, from 2022 to 2023, a total of 438 fresh fecal samples were collected from calves (237 diarrheic and 201 non-diarrheic) (Figure 1). Fecal samples were placed into separate bags marked with the geographical origin, date, age, and sample number, and the type of feces was also recorded and categorized as normal and diarrhea (pasty, watery, or hemorrhagic) according to its physical characteristics. Fresh feces were collected into 15 mL fecal specimen collection tubes immediately from the anus (diarrhea sample) or after defecation by disposable gloves, transported to the lab under cool conditions, and stored in 2.5% potassium dichromate at 4 °C until the extraction of genomic DNA.

### 2.2. DNA Extraction

Each fecal sample was initially washed four times with distilled water and concentrated by centrifugation at 12,000× *g* for 2 min at room temperature. Total DNA was extracted using the E.Z.N.A^®^ Stool DNA kit (Omega Biotek Inc., Norcross, GA, USA) according to the manufacturer’s instructions. The quality and quantity of DNA were determined by electrophoresis in 1% agarose gel and A260/280 with a spectrophotometer (Thermo Fisher Scientific Inc., Waltham, MA, USA), and the extracted DNA was stored at −20 °C for future use.

### 2.3. PCR Amplification

The identification of *Cryptosporidium* spp. was performed by nested PCR amplification of approximately 830 bp of the small-subunit ribosomal RNA (SSU rRNA) gene [23]. The identification of G. duodenalis was performed by nested PCR amplification of approximately 532 bp of the bg gene [24]. The identification of E. bieneusi was performed by nested PCR amplification of approximately 392 bp of their internal transcribed spacer (ITS) of the rRNA gene [25].

The amplification was performed in a 25 μL PCR reaction mixture containing 1 μL extracted genomic DNA (for the primary PCR) or 1 μL of the first PCR amplification product (for the secondary PCR), 2.5 μL 10× Ex Taq Buffer (Mg^2+^ free), 2 mM MgCl_2_, 0.2 mM dNTP Mixture, 0.625 U of TaKaRa Ex Taq (TaKaRa Shuzo Co., Ltd., Kyoto, Japan), and 0.4 μM of each primer. The secondary PCR products were subjected to electrophoresis in 1% (*w*/*v*) agarose gels.

### 2.4. Sequencing and Statistical Analysis

All secondary PCR products were sent to Shanghai Sangon Biological Engineering Biotechnology Company using secondary PCR primers for PCR amplification. If the sequencing results were unclear, the experiment was repeated until a clear result was obtained. All sequences were aligned by the software CLUSTAL X 2.1 (http://www.clustal.org/, accessed on 26 April 2023). To identify different species, subtypes, or genotypes, processed sequences were aligned with the reference sequences in GenBank using BLAST (http://blast.ncbi.nlm.nih.gov, accessed on 26 April 2023). All statistical analyses were performed using SPSS version 22.0 version (SPSS Inc., Chicago, IL, USA). The difference was considered significant when the *p*-value was <0.05. The confidence interval of the proportion was calculated by the Wilson method.

### 2.5. Nucleotide Sequence Accession Numbers

All nucleotide sequences obtained in this study were deposited in the GenBank database under the accession numbers: accession numbers: OQ940362–OQ940371 for the 18S rRNA gene of *Cryptosporidium* spp., OQ978937–OQ978940 for the *bg* gene of *G. duodenalis* and OQ943954–OQ943957 for the ITS gene of *E. bieneusi*.

## 3. Results

### 3.1. The Prevalence of Cryptosporidium spp., G. duodenalis and E. bieneusi in Diarrheic and Non-Diarrheic Calves

PCR amplification of 438 fecal samples showed 46.8% (205/438), 16.9% (74/438), and 10.0% (44/438) of the calves were positive for *Cryptosporidium* spp., *G. duodenalis,* and *E. bieneusi*, respectively. In addition, diarrheic calves demonstrated a significantly higher prevalence of infection with *Cryptosporidium* spp. (χ^2^ = 15.22, *p* < 0.01) compared to non-diarrheic calves (Table 1). In contrast, both the infection rates of *G. duodenalis* and *E. bieneusi* in non-diarrheic calves were higher than those in diarrheic calves. For *G. duodenalis*, non-diarrheic calves had a higher infection rate (18.4%, 37/201) than diarrheic calves (15.6%, 37/237) (χ^2^ = 0.61, *p* > 0.05). Similarly, the prevalence of *E. bieneusi* in non-diarrheic calves was 11.9% (24/201), which was higher than the prevalence in diarrheic calves, which was 8.4% (20/237) (χ^2^ = 1.48, *p* > 0.05).

### 3.2. Age Distribution of Cryptosporidium spp., G. duodenalis and E. bieneusi in Calves

The prevalence of *Cryptosporidium* spp. infection in calves decreases with increasing age, with 54.1% (132/244), 40.0% (52/130), and 32.8% (21/64) for calves <20 d, 20–39 d, and 40–60 d, respectively, and the difference was statistically significant (χ^2^ = 12.66, *p* < 0.01) (Table 2). The prevalence of *G. duodenalis* and *E. bieneusi* infection also differed significantly among calves of different ages (Table 2). Alternatively, the prevalence of *G. duodenalis* and *E. bieneusi* infection in calves increased with increasing age. The prevalence of *G. duodenalis* infection in calves <20 d, 20–39 d, and 40–60 d was 11.1% (27/244), 20.8% (27/130), and 31.3% (20/64), respectively; the prevalence of *E. bieneusi* infection in calves <20 d, 20–39 d, and 40–60 d was 4.1% (10/244), 12.3% (16/130), and 29.7% (19/64), respectively.

Further analysis of the distribution of *Cryptosporidium* spp., *G. duodenalis,* and *E. bieneusi* in diarrheic and non-diarrheic calves of different age groups showed that *Cryptosporidium* spp. infection was found to be associated with diarrhea only in calves aged <20 d (χ^2^ = 7.11, *p* < 0.01); however, *G. duodenalis* and *E. bieneusi* infections in all age groups of calves were not associated with diarrhea (Table 3).

### 3.3. Season Distribution of Cryptosporidium spp., G. duodenalis and E. bieneusi in Calves

In the seasonal groups, the prevalence of *Cryptosporidium* spp. and *G. duodenalis* was significantly different among different seasons. However, there was no significant difference in the prevalence of *E. bieneusi* among different seasonal groups (χ^2^ = 3.69, *p* > 0.05). Consistently, the prevalences of *Cryptosporidium* spp., *G. duodenalis*, and *E. bieneusi* were all highest in autumn with 52.0%, 22.3%, and 11.7%, respectively; all were lowest in summer, with 20.0%, 4.3%, and 4.3%, respectively (Table 4).

Further analysis of the distribution of *Cryptosporidium* spp. in diarrheic and non-diarrheic calves in different seasonal groups revealed that the prevalence of *Cryptosporidium* infection was significantly higher in diarrheic calves than in non-diarrheic calves in autumn and winter, while no significant differences were found in spring and summer (Table 5).

### 3.4. Molecular Analysis of Cryptosporidium spp., G. duodenalis, and E. bieneusi in Ningxia, China

**Figure 2:**
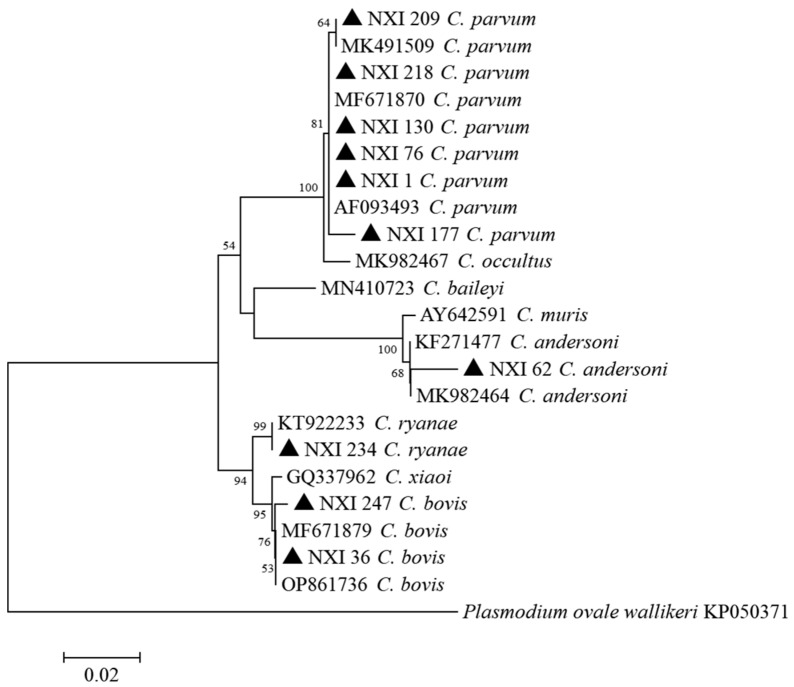
Phylogenetic tree generated by the Neighbor-Joining method using sequences of the SSU rRNA gene of *Cryptosporidium* spp. in the present study and known genotypes previously published in GenBank. The Kimura 2 parameter method was used with bootstrap evaluation of 1000 replicates. The sequences obtained in this study are marked with filled triangles.

**Figure 3:**
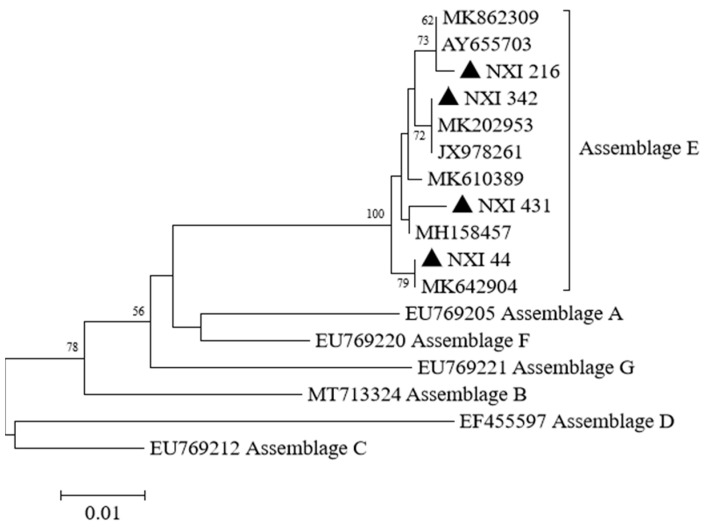
Phylogenetic tree generated by the Neighbor-Joining method using sequences of the *bg* gene of *G. duodenalis* in the present study and known genotypes previously published in GenBank. The Kimura 2 parameter method was used with bootstrap evaluation of 1000 replicates. The sequences obtained in this study are marked with filled triangles.

Among the 205 *Cryptosporidium* spp.-positive samples sequenced in this study, four known *Cryptosporidium* species were successfully identified by comparing with SSU rRNA gene sequences, including *C. parvum*, *C. bovis*, *C. ryanae*, and *C. andersoni* (Figure 2). In addition, all 74 *G. duodenalis*-positive samples were identified as assemblage E by comparative analysis of *bg* gene sequences (Figure 3).

**Figure 4:**
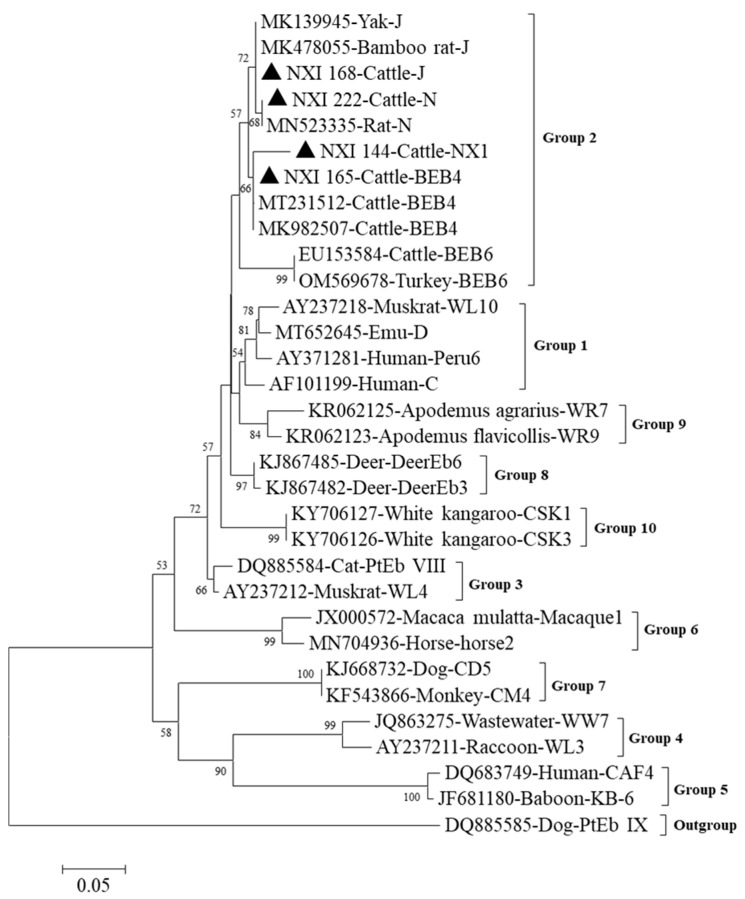
Phylogenetic tree generated by the Neighbor-Joining method using sequences of the ITS region of *E. bieneusi* in the present study and known genotypes previously published in GenBank. The Kimura 2 parameter method was used with bootstrap evaluation of 1000 replicates. The sequences obtained in this study are marked with filled triangles.

Among the 44 *E. bieneusi*-positive samples sequenced in the present study, 4 distinct *E. bieneusi* genotypes were successfully identified by comparative analysis of ITS sequences, including 3 which were known genotypes (J, BEB4, and N) and 1 novel genotype, which was identified and designated as NX1. Furthermore, all four genotypes were found in diarrheic calves, while only one genotype, genotype J, was found in non-diarrheic calves. Phylogenetic analysis indicated that the novel genotype NX1 and the other three known genotypes all belonged to group 2, which has the risk of zoonotic spread (Figure 4).

## 4. Discussion

Cattle are considered to be important zoonotic hosts for *Cryptosporidium* spp., *G. duodenalis*, and *E. bieneusi*, especially calves, which are more vulnerable to parasites. In the present study, the infection rate of *Cryptosporidium* spp. in calves was 48.9% (205/438), which is higher than those reported by most previous studies, for example, rates reported in Ghana (47.8%, 86/180) [26], Western Iran (5.0%, 8/181) [27], and Madagascar (3.5%, 30/862) [28]. These differences could be related to the fact that all fecal samples used in this study were from calves. Past studies have shown that this parasite affects calves and weaned calves more than adults [26,29,30]. The correlation between age and *Cryptosporidium* spp. infection was also confirmed in the present study, where the prevalence of *Cryptosporidium* spp. infection in calves at 20 d, 20–39 d, and 40–60 d was 54.1% (132/244), 40.0% (52/130), and 32.8% (21/64), respectively, and the prevalence decreased with increasing calf age.

Another reason for the higher occurrence rates of *Cryptosporidium* spp. in this study could be that more than half of the calves investigated had diarrhea (54.1%, 237/438). Cattle with diarrhea generally have higher rates of *Cryptosporidium* spp. infection [12,31]. Similarly, in this study, the infection rate of *Cryptosporidium* spp. in diarrheic calves (54.0%, 128/237) was significantly higher than that in non-diarrheic calves (38.3%, 77/201). In this study, four species of *Cryptosporidium* were identified in calves, *C. parvum*, *C. bovis*, *C. ryanae*, and *C. andersoni*. Additionally, *C. parvum* was the predominant species in calves in the study area (83.4%, 171/205), which is consistent with some recent findings of the dominance of *C. parvum* in dairy cattle in Ghana and Latvia [26,32].

Recently, a large number of studies have reported the prevalence of *G. duodenalis* in cattle in China, with a prevalence from 1.0% to 27.5% [16,33,34,35,36,37]. Moreover, in many other countries, such as Turkey, Argentina, and Korea, *G. duodenalis* infections in cattle have also been reported recently with a prevalence of 4.4–41.9% [15,38,39]. The overall *G. duodenalis* prevalence in calves in this study was 16.9% (74/438), which is similar to recent national and international reports. The difference may be related to differences in study design, breed and age of cattle studied, and sampling size, especially the age of the cattle. A recent study using the meta-analysis of the global prevalence of *G. duodenalis* in cattle showed that a positive association was observed between *G. duodenalis* infection with pre-weaned calves (less than 6 months of age) and cattle suffering from diarrhea [40]. Although there was a correlation between the prevalence of *G. duodenalis* infection in calves and age in this study, this correlation was reversed, the prevalence of *G. duodenalis* infection in calves increased with increasing age. *G. duodenalis* infection in calves <20 d, 20–39 d, and 40–60 d was 11.1% (27/244), 20.8% (27/130), and 31.3% (20/64), respectively. More interestingly, in the present study, diarrhea was also not correlated with the prevalence of *G. duodenalis*; on the contrary, the prevalence of infection was higher in non-diarrheic calves than in diarrheic calves, although the difference was not statistically significant. This may be due to the younger age of the calves selected for this study, with the oldest calf in this study being only 60 d. There are relatively few reports on the prevalence of *G. duodenalis* in calves at a younger age, and we need more research data to uncover the regular characteristics of the prevalence of *G. duodenalis*.

To date, eight assemblages (A–H) of *G. duodenalis* have been identified in different hosts [41]. Previous studies have found that assemblage E of *G. duodenalis* is a preponderant assemblage in cattle [40,42], which was further confirmed in this study. In this study, all isolates at the *bg* loci were identified in assemblage E, which is not considered to be zoonotic because it is mainly detected in sheep, goats, and pigs [19]. However, some reports also found assemblage E isolates of *G. duodenalis* in humans, which indicated its zoonotic potential [43,44].

The present study showed that the infection rate of *E. bieneusi* in calves from Ningxia was 10.0% (44/438), which is similar to that reported in other studies for cattle in China and several other countries [45,46,47,48,49]. On the other hand, in the present study, although there was no certain association between diarrhea and the prevalence of *E. bieneusi* infection, *E. bieneusi* genotypes were more diverse in diarrheic calves than in non-diarrheic calves, with all four genotypes found in diarrheic calves, while only the J genotype was found in non-diarrheic calves. Based on the high diversity of ITS, many genotypes of *E. bieneusi* have been found in cattle worldwide [45]. In earlier studies, phylogenetic analyses revealed the existence of 11 groups; genotypes in groups 1 and 2 were associated with potential zoonotic or cross-species infections [18]. Environmental and host isolation characteristics can lead to differences in the distribution of *E. bieneusi* genotypes [50]. In this study, phylogenetic analysis showed that three known genotypes and one novel genotype were clustered into group 2. Thus, all four *E. bieneusi* genotypes identified in cattle in this study might have zoonotic potential.

Diarrhea in newborn calves is a major problem in the cattle industry worldwide, leading to antimicrobial use and mortality, and bringing huge economic losses. Parasites are one of the main pathogens of cattle diarrhea, especially in calves [11]. From these observations in this study, it appears that *Cryptosporidium* spp. is more harmful to calves than *G. duodenalis* and *E. bieneusi*, not only because of its higher infection rate, but also because of its significant correlation with diarrhea in calves, especially in calves within 20 days of age. This information can be taken into consideration by health policymakers, veterinarians, and farmers for the scientific management of newborn calves.

## 5. Conclusions

The present study revealed the high occurrence of *Cryptosporidium* spp., *G. duodenalis*, and *E. bieneusi* in calves in Ningxia, China. The total prevalence of *Cryptosporidium* spp. *G. duodenalis*, and *E. bieneusi* infection were 46.8%, 16.9%, and 10.0%, respectively. *Cryptosporidium* spp. infection was closely related to diarrhea of calves within 20 days of age. Four Cryptosporidium species (*C. parvum*, *C. bovis*, *C. ryanae*, and *C. andersoni*), an assemblage E of *G. duodenalis*, and four *E. bieneusi* genotypes (BEB4, N, J, and NX1) were observed. The findings in our study provided basic data for understanding the molecular epidemiology of *Cryptosporidium* spp., *G. duodenalis*, and *E. bieneusi* in diarrheic and non-diarrheic calves, and unraveling the intricate molecular epidemiology of Cryptosporidiosis, microsporidiosis, and giardiasis in calves and its impact on human health.

## Figures and Tables

**Table 1:** The prevalence of parasitic infections in diarrheic and non-diarrheic calves.

Species	No. Positive (%, 95%CI)		χ^2^ (*p*-Value)
	Diarrheic	Non-Diarrheic	
*Cryptosporidium* spp.	128 (54.0, 47.7–60.2)	77 (38.3, 31.9–45.2)	χ^2^ = 15.22, *p* < 0.01
*G. duodenalis*	37 (15.6, 11.5–20.8)	37 (18.4, 13.7–24.3)	χ^2^ = 0.61, *p* = 0.436
*E. bieneusi*	20 (8.4, 5.5–12.7)	24 (11.9, 8.2–17.2)	χ^2^ = 1.48, *p* = 0.224

**Table 2:** Prevalence and identification of *Cryptosporidium* spp., *G. duodenalis* and *E. bieneusi* in calves of different ages.

Species	Age	No. Positive/Examined (%, 95%CI)	χ^2^ (*p*-Value)	Species/Assemblages/Genotypes (No.)	
				Diarrheic	Non-Diarrheic
*Cryptosporidium* spp.	<20 d	132/244 (54.1, 47.8–60.2)	χ^2^ = 12.66, *p* < 0.01	*C. parvum* (92), *C. bovis* (8), *C. andersoni* (1)	*C. parvum* (30), *C. andersoni* (1)
	20–39 d	52/130 (40.0, 32.0–48.6)		*C. parvum* (16), *C. bovis* (4), *C. andersoni* (4)	*C. parvum* (23), *C. bovis* (3), *C. andersoni* (2)
	40–60 d	21/64 (32.8, 22.6–45.0)		*C. parvum* (2), *C. bovis* (1)	*C. parvum* (8), *C. bovis* (5), *C. andersoni* (4), *C. ryanae* (1)
*G. duodenalis*	<20 d	27/244 (11.1, 7.7–15.6)	χ^2^ = 16.69, *p* < 0.01	E (18)	E (9)
	20–39 d	27/130 (20.8, 14.7–28.5)		E (11)	E (16)
	40–60 d	20/64 (31.3, 21.2–43.4)		E (8)	E (12)
*E. bieneusi*	<20 d	8/244 (3.3, 1.7–6.3)	χ^2^ = 41.01, *p* < 0.01	J (5), NXI (1), BEB4 (1)	J (1)
	20–39 d	17/130 (13.1, 8.3–20.0)		J (7), BEB4 (1)	J (9)
	40–60 d	19/64 (29.7, 19.9–41.8)		J (3), N (1), BEB4 (1)	J (14)

**Table 3:** Prevalence of *Cryptosporidium* spp., *G. duodenalis,* and *E. bieneusi* in diarrheic and non-diarrheic calves of different ages.

Species	Age	No. Positive/Examined (%, 95%CI)		χ^2^ (*p*-Value)
		Diarrheic	Non-Diarrheic	
*Cryptosporidium* spp.	<20 d	101/169 (59.8, 52.2–66.9)	31/75 (41.3, 30.9–52.6)	χ^2^ = 7.11, *p* < 0.01
	20–39 d	24/51 (47.1, 34.1–60.5)	28/79 (35.4, 25.8–46.4)	χ^2^ = 1.74, *p* > 0.05
	40–60 d	3/17 (17.7, 6.2–41.0)	18/47 (38.3, 25.8–52.6)	χ^2^ = 2.42, *p* > 0.05
*G. duodenalis*	<20 d	18/169 (10.7, 6.8–16.2)	9/75 (12.0, 6.4–21.3)	χ^2^ = 0.10, *p* > 0.05
	20–39 d	11/51 (21.6, 12.5–34.6)	16/79 (20.3, 12.9–30.4)	χ^2^ = 0.03, *p* > 0.05
	40–60 d	8/17 (47.1, 26.2–69.0)	12/47 (25.5, 15.3–39.5)	χ^2^ = 2.69, *p* > 0.05
*E. bieneusi*	<20 d	7/169 (4.1, 2.0–8.3)	1/75 (1.3, 0.2–7.2)	Fisher’s Test ^1^, *p* > 0.05
	20–39 d	8/51 (15.7, 8.2–28.0)	9/79 (11.4, 6.1–20.3)	χ^2^ = 0.50, *p* > 0.05
	40–60 d	5/17 (29.4, 13.3–53.1)	14/47 (29.8, 18.7–44.0)	χ^2^ = 0.001, *p* > 0.05

^1^ Fisher’s exact test was applied here because, in this group of data, the data matrix presented that one of the four cells (25%) had an expected count of less than five; the same table below.

**Table 4:** Prevalence and identification of *Cryptosporidium* spp., *G. duodenalis,* and *E. bieneusi* in calves in different seasons.

Species	Season	No. Positive/Examined (%, 95%CI)	χ^2^ (*p*-Value)	Species/Assemblages/Genotypes	
				Diarrheic	Non-Diarrheic
*Cryptosporidium* spp.	Spring	31/60 (51.7, 39.3–63.4)	χ^2^ = 24.04, *p* < 0.01	*C. parvum* (21), *C. bovis* (2)	*C. parvum* (8)
	Summer	14/70 (20.0, 12.3–30.8)		*C. parvum* (6), *C. andersoni* (1)	*C. parvum* (7)
	Autumn	133/256 (52.0, 45.9–58.0)		*C. parvum* (70), *C. bovis* (9)	*C. parvum* (45), *C. bovis* (7), *C. ryanae* (1), *C. andersoni* (1)
	Winter	27/52 (51.9, 38.7–64.9)		*C. parvum* (13), *C. bovis* (2), *C. andersoni* (4)	*C. parvum* (1), *C. bovis* (1), *C. andersoni* (6)
*G. duodenalis*	Spring	6 /60 (10.0, 4.7–20.2)	χ^2^ = 15.30, *p* < 0.01	E (2)	E (4)
	Summer	3/70 (4.3, 1.5–11.9)		E (3)	/
	Autumn	57/256 (22.3, 17.6–27.8)		E (28)	E (29)
	Winter	8/52 (15.4, 8.0–27.5)		E (4)	E (4)
*E. bieneusi*	Spring	5/60 (8.3, 3.6–18.1)	χ^2^ = 3.69, *p* > 0.05	J (3)	J (2)
	Summer	3/70 (4.3, 1.5–11.9)		J (2)	J (1)
	Autumn	30/256 (11.7, 8.3–16.2)		J (7), NXI (1), BEB4 (3)	J (19)
	Winter	6/52 (11.5, 5.4–23.0)		J (3), N (1)	J (2)

**Table 5:** Prevalence of *Cryptosporidium* spp., *G. duodenalis,* and *E. bieneusi* in diarrheic and non-diarrheic calves in different seasons.

Species	Season	No. Positive/Examined (%, 95%CI)		χ^2^ (*p*-Value)
		Diarrheic	Non-Diarrheic	
*Cryptosporidium* spp.	Spring	23/38 (60.5, 44.7–74.4)	8/22 (36.4, 19.7–57.0)	χ^2^ = 3.26, *p* > 0.05
	Summer	7/35 (20.0, 10.0–35.9)	7/35 (20.0, 10.0–35.9)	χ^2^ = 0.00, *p* > 0.05
	Autumn	79/136 (58.1, 49.7–66.1)	54/120 (45.0, 36.4–53.9)	χ^2^ = 4.38, *p* < 0.05
	Winter	19/28 (67.9, 49.3–82.1)	8/24 (33.3, 18.0–53.3)	χ^2^ = 6.17, *p* < 0.05
*G. duodenalis*	Spring	2/38 (5.3, 1.5–17.3)	4/22 (18.2, 7.3–38.5)	Fisher’s Test, *p* > 0.05
	Summer	3/35 (8.6, 3.0–22.4)	0/35 (0.0, 0.0–9.9)	Fisher’s Test, *p* > 0.05
	Autumn	28/136 (20.6, 14.7–28.2)	29/120 (24.2, 17.4–32.6)	χ^2^ = 4.72, *p* > 0.05
	Winter	4/28 (14.3, 5.7–31.5)	4/24 (16.7, 6.7–35.9)	Fisher’s Test, *p* > 0.05
*E. bieneusi*	Spring	3/38 (7.9, 2.7–20.8)	2/22 (9.1, 2.5–27.8)	Fisher’s Test, *p* > 0.05
	Summer	2/35 (5.7, 1.6–18.6)	1/35 (2.9, 0.5–14.5)	Fisher’s Test, *p* > 0.05
	Autumn	11/136 (8.1, 4.6–13.9)	19/120 (15.8, 10.4–23.4)	χ^2^ = 3.70, *p* > 0.05
	Winter	4/28 (14.3, 5.7–31.5)	2/24 (8.3, 2.3–25.8)	Fisher’s Test, *p* > 0.05

## Data Availability

Data are contained within the article.
